# Environmentally Friendly Compatibilizers from Soybean Oil for Ternary Blends of Poly(lactic acid)-PLA, Poly(ε-caprolactone)-PCL and Poly(3-hydroxybutyrate)-PHB

**DOI:** 10.3390/ma10111339

**Published:** 2017-11-22

**Authors:** María Jesús Garcia-Campo, Luis Quiles-Carrillo, Jaime Masia, Miguel Jorge Reig-Pérez, Nestor Montanes, Rafael Balart

**Affiliations:** 1 Institute of Materials Technology (ITM), Universitat Politècnica de València (UPV), Plaza Ferrándiz y Carbonell 1, 03801 Alcoy, Alicante, Spain; mjgcampo@gmail.com (M.J.G.-C.); luiquic1@epsa.upv.es (L.Q.-C.), mjreig@mcm.upv.es (M.J.R.-P.); nesmonmu@upvnet.upv.es (N.M.); 2 Institute of Design and Manufacturing (IDF), Universitat Politècnica de València (UPV), Plaza Ferrándiz y Carbonell 1, 03801 Alcoy, Alicante, Spain; jmasia@mcm.upv.es

**Keywords:** PLA, toughness, ternary blend, compatibilization, vegetable oils, environmentally friendly

## Abstract

Ternary blends of poly(lactic acid) (PLA), poly(3-hydroxybutyrate) (PHB) and poly(ε-caprolactone) (PCL) with a constant weight percentage of 60%, 10% and 30% respectively were compatibilized with soybean oil derivatives epoxidized soybean oil (ESO), maleinized soybean oil (MSO) and acrylated epoxidized soybean oil (AESO). The potential compatibilization effects of the soybean oil-derivatives was characterized in terms of mechanical, thermal and thermomechanical properties. The effects on morphology were studied by field emission scanning electron microscopy (FESEM). All three soybean oil-based compatibilizers led to a noticeable increase in toughness with a remarkable improvement in elongation at break. On the other hand, both the tensile modulus and strength decreased, but in a lower extent to a typical plasticization effect. Although phase separation occurred, all three soybean oil derivatives led somewhat to compatibilization through reaction between terminal hydroxyl groups in all three biopolyesters (PLA, PHB and PCL) and the readily reactive groups in the soybean oil derivatives, that is, epoxy, maleic anhydride and acrylic/epoxy functionalities. In particular, the addition of 5 parts per hundred parts of the blend (phr) of ESO gave the maximum elongation at break while the same amount of MSO and AESO gave the maximum toughness, measured through Charpy’s impact tests. In general, the herein-developed materials widen the potential of ternary PLA formulations by a cost effective blending method with PHB and PCL and compatibilization with vegetable oil-based additives.

## 1. Introduction

In the last decade poly(lactic acid) (PLA) has become one of the most promising biopolymers due to its balanced properties (mechanical, thermal, barrier, etc.) together with a cost competitive price and easy processing by conventional techniques. For these reasons, PLA finds increasing uses in a wide range of industrial sectors such as automotive [1,2,3,4], medical devices [5,6], construction and building, 3D printing [7,8], packaging [9,10,11,12], wood plastic composites (WPCs) [13,14], and so on. Nevertheless, PLA is a very fragile polymer with very low toughness. With the aim of increasing its toughness, important research has been carried out in the last decade. These research works have focused on different approaches to overcome this important drawback for most industrial uses. One approach is the use of plasticizers such as poly(ethylene glycol) (PEG) [15], acetyl tributyl citrate (ATBC) [16,17], triethyl citrate (TEC) [18], lactic acid oligomers [19,20,21], and so on. This solution is interesting as an important elongation at break is achieved, but other mechanical resistant properties (modulus, strength) are remarkably reduced. Another approach is copolymerization, for example, poly(lactide)-*g*-poly(butylene succinate-*co*-adipate) [22], but it is not a cost-effective solution. The third approach is blending PLA with other polymers to obtain toughened formulations. There are many works focused on binary blends of PLA with poly(ε-caprolactone) (PCL) [23,24], poly(hydroxyalkanoates) (PHAs) [9,10,25], thermoplastic starch (TPS) [26,27], poly(butylene succinate) (PBS) [28,29], poly(butylene succinate-*co*-adipate) (PBSA) [30], poly(butylene adipate-*co*-terephthalate) (PBAT) [31], cellulose [32], and so on. Due to the low miscibility of PLA with most polymers, the use of compatibilizers and/or reactive extrusion have been proposed as technical solutions to improve compatibility in binary PLA-based blends [30,33]. Moreover, ternary blends with PLA have also given good results in terms of improved toughness as tailored properties can be obtained by selecting the appropriate components and/or compatibilizers [24,34,35,36,37,38,39].

This search for high environmentally friendly tough PLA formulations also includes the use of low environmental impact additives, for example, plasticizers, compatibilizers, chain extenders, and so on. Vegetable oils (VOs) represent an interesting alternative to petroleum-derived polymers and additives [40,41,42]. VOs are composed of a triglyceride structure in which, three fatty acids are linked to a glycerol-base structure through ester bonds. Some fatty acids are very interesting from a chemical point of view due to presence of one or several unsaturations. These unsaturations allow different chemical modifications that can be used to tailor the desired properties [43]. Among all fatty acids, oleic, linoleic and linolenic acids, which contain one, two and three carbon-carbon double bonds respectively, play a key role in VO functionalization. There is a wide variety of VOs but industrially, linseed oil (LO) and soybean oil (SO) represent a high volume market. Today, it is possible to find epoxidized, maleinized and acrylated-epoxidized vegetable oils at industrial scale with different purposes. Epoxidized soybean oil (ESO) and epoxidized linseed oil (ELO) find interesting uses in the poly(vinyl chloride) (PVC) plasticization industry, as well as in the thermosetting industry as base resins for green composites. Torres-Giner et al. reported new PVC wood flour composites with epoxidized linseed oil (ELO) which played a dual role in plasticization and polymer-particle compatibilization [44]. The plasticization effect of epoxidized vegetable oils has also been studied in different biopolyesters, such as PLA and PHB, leading to a slight decrease in the glass transition temperature but important improvements on toughness [45,46,47]. Due to the high reactivity of the oxirane ring towards hydroxyl groups, epoxidized vegetable oils have also been used as compatibilizers in composite materials [48]. Samper et al. reported the potential of ELO-based composites with basalt fibers as substitutes of glass fiber composites, using different crosslinking agents [49,50]. Acrylated epoxidized soybean oil (AESO) finds increasing uses in partially biobased thermosetting acrylic and unsaturated polyester resins. Different research works have focused on the development of thermosetting resins with AESO directly [51] or with different copolymers, such as methacrylated eugenol [52], N-vinyl-2-pyrrolidone [53], isosorbide methacrylate [54], rosin-based acrylamide [55] and so on, with potential uses in composites. AESO has also been reported as additive in toughened PLA formulations [37]. Maleinized linseed oil has been proposed for a wide variety of industrial applications which include base for soluble resins, biolubricant formulations, wood treatments, base for vegetable polyols, among others. Carbonell-Verdu et al. reported a dual effect of maleinized cottonseed oil (MCSO) on PLA films: on one hand, some plasticization occurred and on the other hand, a chain extension/branching phenomenon was observed [56]. This chain extension effect was also reported by Ernzen et al. with polyamide 6 [57]. Ferri et al. also reported a compatibilization effect of maleinized linseed oil (MLO) on PLA blends with thermoplastic starch [58], and a clear improvement on toughness of neat PLA [59]. This positive effect of a maleinized vegetable oil on toughness was also found for poly(3-hydroxybutyrate) (PHB) as suggested by Garcia-Garcia et al. [60] In addition to its potential in the polymer industry, aqueous solutions of maleinized soybean oil (MSO) have been successfully used as formaldehyde-free coatings in textiles as reported by Ford et al. [61].

This work explores the potential of soybean oil derivatives, namely epoxidized soybean oil (ESO), epoxidized-acrylated soybean oil (AESO) and maleinized soybean oil (MSO) as an environmentally friendly solution to increase the compatibility of toughened poly(lactic acid) (PLA) formulations via melt blending with two other polyesters, namely poly(3-hydroxybutyrate) (PHB) and poly(ε-caprolactone) (PCL). The functionalities present in ESO, AESO and MSO (epoxy, acrylic acid and maleic anhydride respectively) could react with the hydroxyl terminal groups in all three polyesters thus leading to a compatibilization effect. The results are compared with a petroleum-based epoxy styrene-acrylic oligomer (ESAO) to assess the potential of the soybean oil derivatives for industrial formulations. This oligomer is widely used with individual polyesters as chain extender to overcome the effects of hydrolysis during processing at high temperatures and, in addition, it is also used as compatibilizer in polyester blends as its functional groups (acrylic, epoxy) can react with hydroxyl groups in biopolyesters. For these reasons, ESAO has been selected as control material to compare the effectiveness of the soybean oil-based compatibilizers.

## 2. Results and Discussion

### 2.1. Effect of Soybean-Derived Compatibilizers on Mechanical Properties and Morphology of Ternary PLA/PHB/PCL Blends

Table 1 summarizes the mechanical properties of the ternary PLA_60_/PHB_10_/PCL_30_ blend with different additives. Neat PLA is a quite brittle polymer with an elongation at break of 7.87%. Its tensile modulus and strength are 3.6 GPa and 58.2 MPa respectively. The PLA_60_/PHB_10_/PCL_30_ blend, shows a noticeable increase in elongation at break up to values of 15.3%. Due to the flexible nature of the PCL component, the tensile strength and modulus decrease down to values of 48.4 MPa and 2.0 GPa respectively. It is well known that PCL is highly immiscible with PLA, while PHB (especially low molecular grades) shows some (although restricted) miscibility [62,63,64]. This lack of full miscibility does not allow a continuous phase and subsequently, load transfer between phases is not optimum. To overcome or minimize this phenomenon, compatibilizers are added to blends. As can be seen in Table 1, all three soybean oil-based compatibilizers improve the ductile properties thus giving evidence of some plasticization and/or compatibilization. Similar results were reported by Li et al. in PLA toughened formulations with poly(butadiene) core-shell particles and a multi-functionalized glycidyl/acrylic shell to react with the PLA matrix and partially compatibilize the system [65]. For the PLA_60_/PHB_10_/PCL_30_ blend, the elongation at break increases up to values of 44% with 1 phr conventional petroleum-based multifunctional compatibilizer, ESAO. It is worthy to note that AESO gives similar elongation at break values of 45%, as well as similar tensile strength and modulus, with values of 43–44 MPa and 1.8–1.9 GPa respectively. It has been suggested that the multifunctional oligomer ESAO, as well as the acrylated oil, could lead to compatibilization through reaction with hydroxyl terminal groups in all three biopolyesters in the blend, but also some branching and/or crosslinking could potentially occur. That is why mechanical resistant properties are not remarkably reduced, compared to the uncompatibilized blend [37,66]. It is worth highlighting the high elongation at break that ESO gives to the blend, reaching values of about 130%. In contrast, tensile strength is lower than that provided by ESAO and AESO. This could suggest that the main acting mechanism of ESO is plasticization and slight interphase compatibilization through reaction of oxirane rings with hydroxyl groups in PLA, PHB and PCL. A similar synergistic plasticization plus compatibilization behavior has been observed by Balart et al. on green composites of PLA and hazelnut shell flour with epoxidized linseed oil (ELO). They report a slight plasticization phenomenon with a low decrease in T_g_ and on the other hand, the wetting of the lignocellulosic particles increases in a great extent due to the reaction of ELO with hydroxyl groups present in both PLA and lignocellulosic particles [13]. Finally, addition of 5 phr MSO also leads to a remarkable increase in elongation at break, up to 65.8% with a noticeable decrease in both tensile strength and modulus down to similar values of the blend compatibilized with ESO. Ferri et al. revealed the synergistic effect that maleinized linseed oil (MLO) can provide to binary PLA/TPS blends with 30 wt% TPS. They reported a remarkable increase in elongation at break up to 160% and a clear compatibilization, detectable by SEM observation as well as a decrease in T_g_ by 10 °C with the only addition of 6 phr MLO [58].

With regard to the hardness of the materials developed in this study, the uncompatibilized blend offers a Shore D hardness close to 72. It is worth noting the high Shore D values that AESO-compatibilized blend provides, with values of about 88. This is in agreement with the above-mentioned mechanical properties and it is directly related to the branching/crosslinking phenomenon of AESO. Regarding the impact-absorbed energy obtained by Charpy’s impact tests, the following assessments can be made. Neat PLA (notched sample) shows a very low impact strength of 1.63 kJ m^−2^. The uncompatibilized blend shows a remarkable increase in toughness with an impact strength of 5.06 kJ m^−2^, which is almost five times the value of the neat PLA. This indicates that the physical blending of PLA with PHB and PCL gives a toughen PLA formulation. Nevertheless, the poor compatibility between the three polyesters (mainly between PLA and PCL) does not allow optimum load transfer. As it can be seen in Table 1, all three soybean oil-based compatibilizers provide a remarkable increase in impact strength up to values of almost 11 kJ m^−2^ for the AESO- and MSO-compatibilized blend. These results indicate the positive effect of these additives in compatibilizing the blend.

**Scheme 1:**
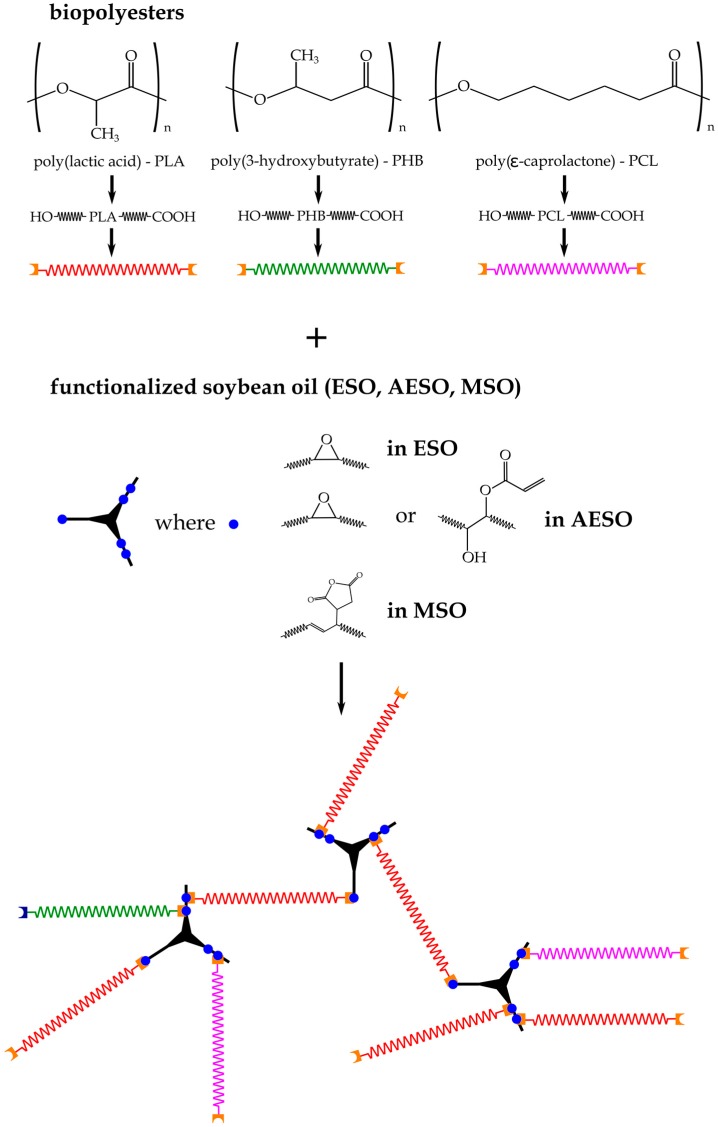
Schematic representation of the possible reactions between the chemically modified soybean oil additives (epoxidized soybean oil (ESO), acrylated epoxidized soybean oil (AESO) and maleinized soybean oil (MSO)) and biopolyesters in ternary blend.

This good mechanical performance is due to two main phenomena. On one hand, the chemically modified soybean oils (with more polar groups) offer a similar solubility parameter to that of biopolyesters, thus allowing interactions between them. This phenomenon mainly leads to a plasticization effect. On the other hand, the functionalities of the soybean oil-based compatibilizers (epoxy in ESO, epoxy and acrylate in AESO, and maleic anhydride in MSO) can readily react with the hydroxyl terminal groups in all three biopolyesters thus leading do several processes such as chain extension, branching and/or crosslinking [67]. These two phenomena (plasticization and chemical reaction) overlaps and the overall effects are positive on mechanical performance as seen in Table 1. Plasticization provides increased elongation at break and reaction (chain extension, branching and/or crosslinking) leads to increased toughness as these reactions are responsible for a low decrease in mechanical resistant properties while the elongation at break (due to the plasticization effect) is remarkably increased. The combination of relatively high mechanical resistant properties, together with a high increase in elongation at break, leads to a noticeable increase in impact strength. The possible reactions of the biopolyesters with the functionalized soybean oil compatibilizers are summarized in Scheme 1.

**Figure 1:**
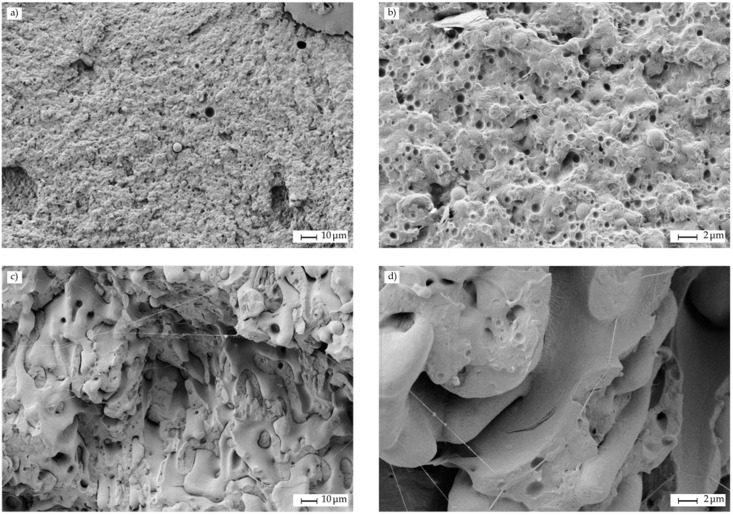

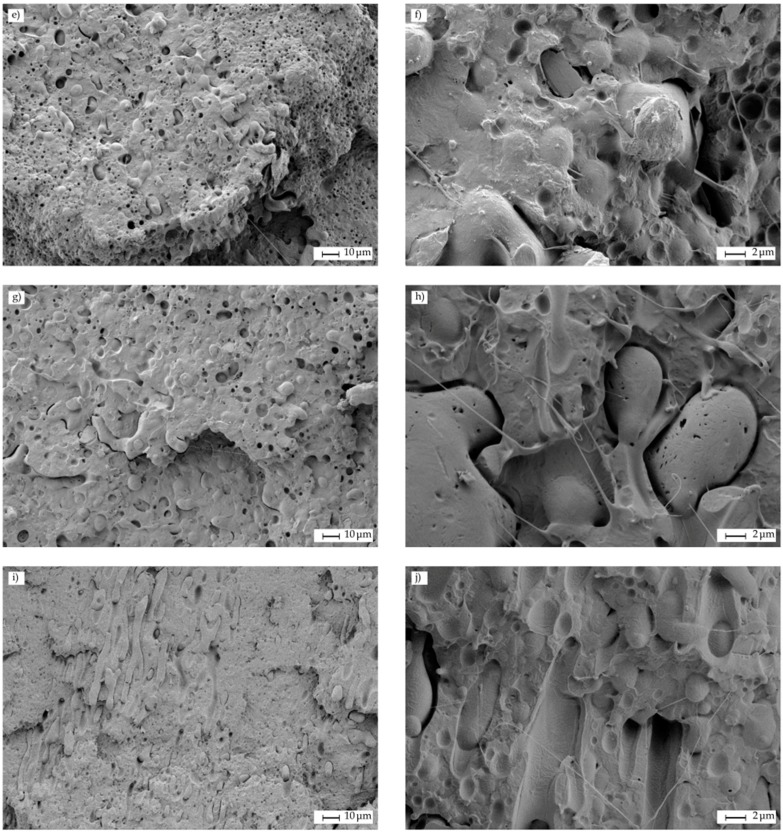
Field emission scanning electron microscopy (FESEM) images of the fractured samples from impact tests corresponding to the PLA_60_/PHB_10_/PCL_30_ blend with different compatibilizers at 500× (left) and 3000× (right). (**a**,**b**) uncompatibilized; (**c**,**d**) compatibilized with 5 phr ESO; (**e**,**f**) compatibilized with 5 phr AESO; (**g**,**h**) compatibilized with 5 phr MSO; (**i**,**j**) compatibilized with 1 phr epoxy styrene-acrylic oligomer (ESAO).

The mechanical behavior of uncompatibilized and compatibilized PLA_60_/PHB_10_/PCL_30_ is directly related to its morphology. Previous results have demonstrated the immiscibility (or very poor) between PLA and PCL [68], as well as the binary system composed of PHB and PCL [69]. In both cases, the simple addition of PCL which appears as randomly dispersed spherical, positively contributes to improve toughness. This increase in toughness is much higher in the PLA/PCL blend than in the PHB/PCL blend as reported by these previous studies. Figure 1 shows the morphology of the fractured samples from impact tests. The uncompatibilized blend shows a clear droplet-like surface morphology with a PLA matrix in which submicron PHB and PCL droplets are finely dispersed. At high magnification, the droplet structure (Figure 1b) is clearly revealed. It is worthy to note the presence of a high number of spherical voids, which are mainly related to removed PCL droplets due to its low compatibility with PLA. The morphology of the blend compatibilized with all three soybean oil-based additives is completely different as it can be observed at both low (left) and high (right) magnification in Figure 1. The morphology of the blend compatibilized with ESO offers quite good phase continuity as well as important signs of plastic deformation during the impact test. Some voids can be detected and full continuity is not achieved but the plasticization effect of the functionalized vegetable oil gives with interactions with all three biopolyesters leads to a different fracture surface (Figure 1c,d). AESO-compatibilized blend shows the typical droplet-like morphology but presence of voids is less intense. This is due to the partial compatibilization effect of AESO with hydroxyl groups that allows more intense interactions between PCL and the surrounding PLA matrix (Figure 1e,f). In fact, it can be clearly seen at high magnification (Figure 1d) that some of the spherical areas, mainly attributable to PCL (as its content is higher than PHB), show a fully wetted surface and this is an important change with regard to uncompatibilized blend. Similar behavior has been observed in ternary blends of PLA, TPS and PCL as indicated by Mittal et al. [23]. MSO-compatibilized blend also shows a change in its morphology, compared to uncompatibilized blend. Although phase separation occurs (Figure 1g) and is evident at low magnification (500×), it is not clearly distinguishable the matrix and the dispersed droplet-like structure at higher magnification (Figure 1h). Similar findings were reported by Ferri et al. in PLA/TPS blends compatibilized with different amounts of maleinized linseed oil (MLO). They also suggested dual effect of MLO: on one hand, a plasticization effect and, on the other hand, compatibilization through reaction with hydroxyl groups in starch and biopolyester [58]. Similar results are provided by a conventional epoxy styrene-acrylic oligomer, ESAO. Although phase separation is still detectable (Figure 1i,j), the typical spherical shapes, change to elongated shapes due to plastic deformation due to the reactivity of epoxy groups towards hydroxyl groups in all three biopolyesters as reported by Torres-Giner et al. with poly(hydroxyalkanoates) and sepiolite composites, compatibilized with this multi-functional epoxy styrene-acrylic oligomer [70].

**Figure 2:**
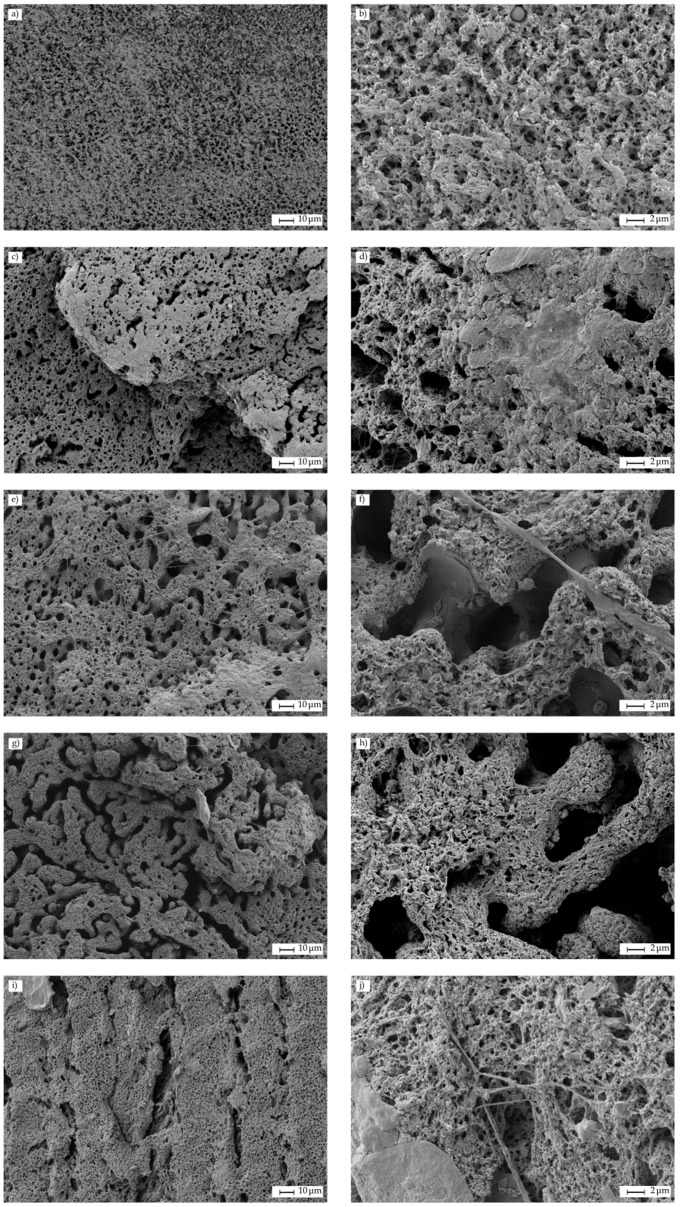
FESEM images of the fractured samples from impact tests corresponding to the PLA_60_/PHB_10_/PCL_30_ blend with different compatibilizers at 500× (left) and 3000× (right), subjected to selective extraction with glacial acetic acid. (**a**,**b**) uncompatibilized; (**c**,**d**) compatibilized with 5 phr ESO; (**e**,**f**) compatibilized with 5 phr AESO; (**g**,**h**) compatibilized with 5 phr MSO; (**i**,**j**) compatibilized with 1 phr ESAO.

The selective extraction with acetic acid mainly affects to PCL and PHB, while the PLA matrix remains almost unaltered as can be seen in Figure 2. Uncompatibilized blend (Figure 2a,b) shows a homogeneous rough surface in which, PCL and PHB have been selectively removed. ESO compatibilizer seems to give the highest homogeneity (Figure 2c,d) while both AESO and MSO seem to give less homogeneity, probably due to heterogeneous mixing with the blend during the extrusion, leading to some highly compatibilized areas that coexist with poorly compatibilized areas. With regard to petroleum-based ESAO, it is worthy to note that seems to offer reduced domain shapes and evidences of plastic deformation are clearly distinguishable after selective extraction (Figure 2i,j).

### 2.2. Effect of Soybean-Derived Compatibilizers on Thermal Properties of Ternary PLA/PHB/PCL Blends

**Figure 3:**
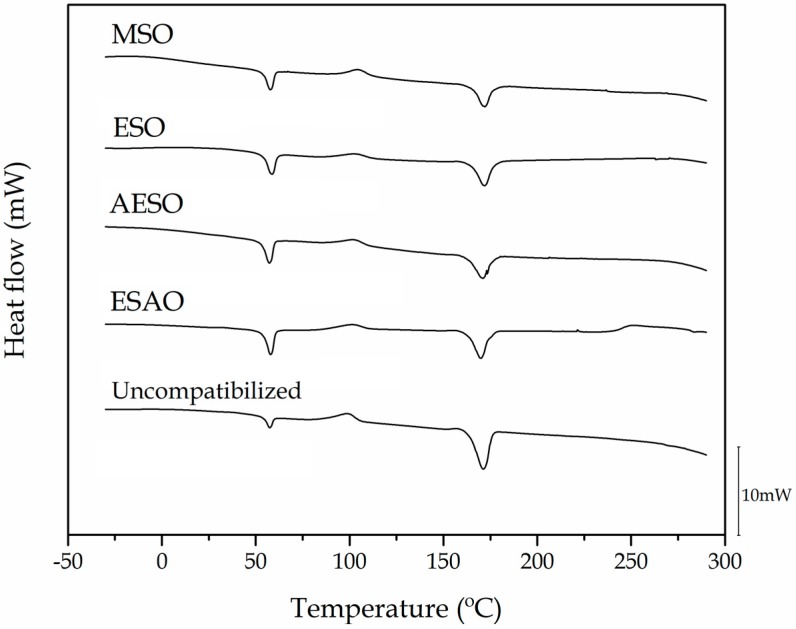
Comparative differential scanning calorimetry (DSC) thermograms (2nd heating) of PLA_60_/PHB_10_/PCL_30_ blend with different compatibilizers.

Figure 3 shows a comparative plot of the differential scanning calorimetry (DSC) thermal profiles of the uncompatibilized and the different compatibilized PLA_60_/PHB_10_/PCL_30_ blend. Due to some overlapping processes it is not possible to distinguish in a clear way the particular thermal transitions of each polymer in the blend and the effects of the soybean oil-based compatibilizers. These overlapping processes are the following: on one hand, the typical glass transition of PLA is located at about 55–60 °C and overlaps with the melt peak of PCL (58–60 °C). On the other hand, the melt temperature range for PLA (155–170 °C) overlaps with the typical melt peak temperature of PHB at about 170 °C. It has also been reported that PHB also affects the cold crystallization process in PLA by restricting chain packing in ordered form [71]. Even with the presence of some compatibilizers, conventional DSC is not sensitive enough to measure the slight changes that all compatibilizers provide to the thermal transitions of biopolyesters. Nevertheless, modulated DSC could be used to clearly separate the main thermal transitions even if they appear overlapped. Conventional DSC without modulated signal is useful to identify the melt peaks of the two main components, PLA and PCL, and know different temperature ranges such as the upper service temperature (below 50 °C) and the processing window for these blends (between 175 and 230 °C to avoid degradation). With regard to the thermal transitions, the use of dynamic mechanical thermal analysis is also useful to identify them in a clear way as it will be discussed later.

**Figure 4:**
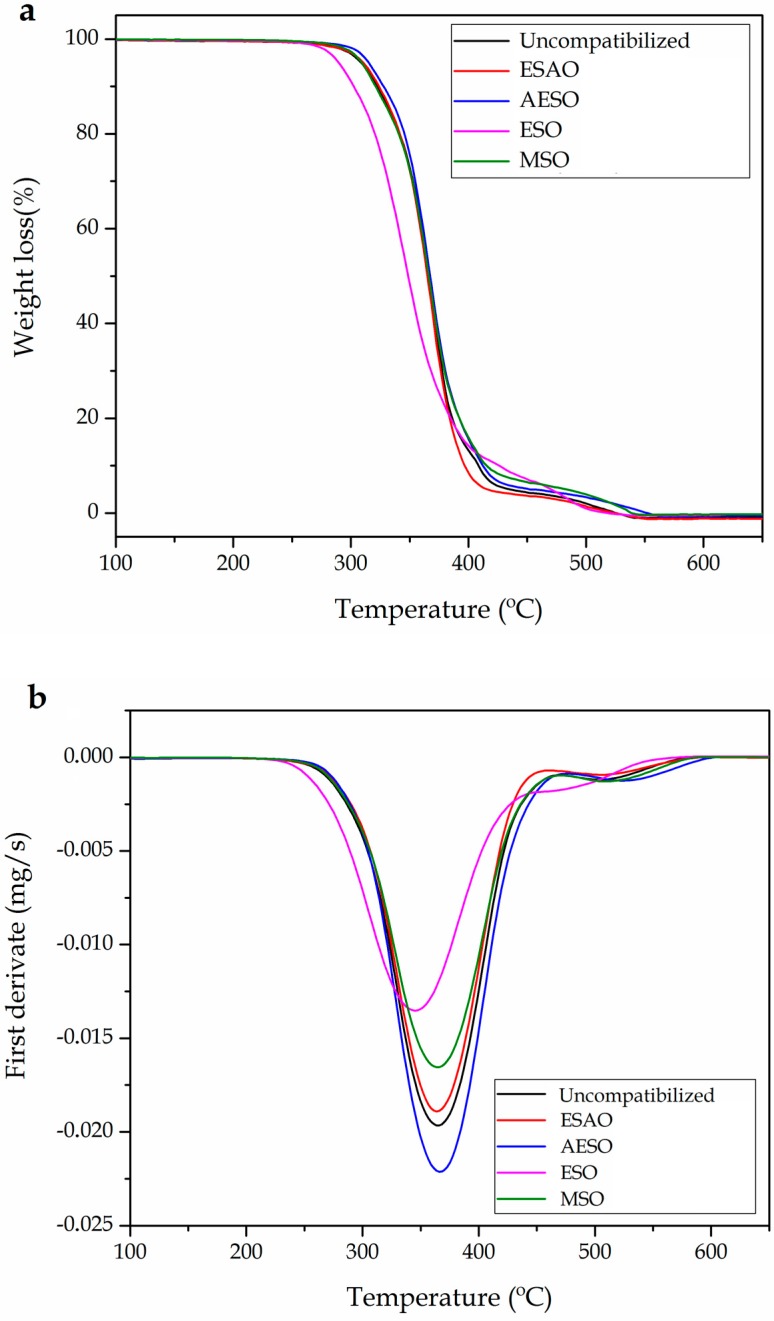
Comparative thermogravimetric (TGA) thermograms of PLA_60_/PHB_10_/PCL_30_ blend with different compatibilizers, (**a**) weight loss and (**b**) DTG first derivative.

With regard to the thermal stability, Figure 4 gathers the thermogravimetric analysis (TGA) and first derivative (DTG) curves for uncompatibilized and compatibilized PLA_60_/PHB_10_/PCL_30_ blend. As can be assessed from the TGA profiles, ELO is the only compatibilizer that provides decreased thermal stability to the blend but, in contrast, it is the compatibilizer which enhances the highest elongation at break values as above mentioned. The onset degradation temperature, measured at a constant weight loss of 5% (T_5_) changes from 308.2 °C (uncompatibilized blend) down to 289.1 °C for the ESO-compatibilized blend (Table 2). This tendency is also observed for the maximum degradation rate temperature (T_max_) which corresponds to the peak value of the DTG curves. In particular, the T_max_ for the uncompatibilized blend is 364.6 °C and decreases by 20 °C for the ESO-compatibilized blend. All other soybean oil-based compatibilizers, including petroleum-based ESAO, do not lead to a remarkable change in the T_max_ values, located at about 364–365 °C. It is worthy to note the stabilizing effect of AESO in the initial stages of the degradation process, which could be related to the branching/crosslinking effect that AESO can provide [37].

**Figure 5:**
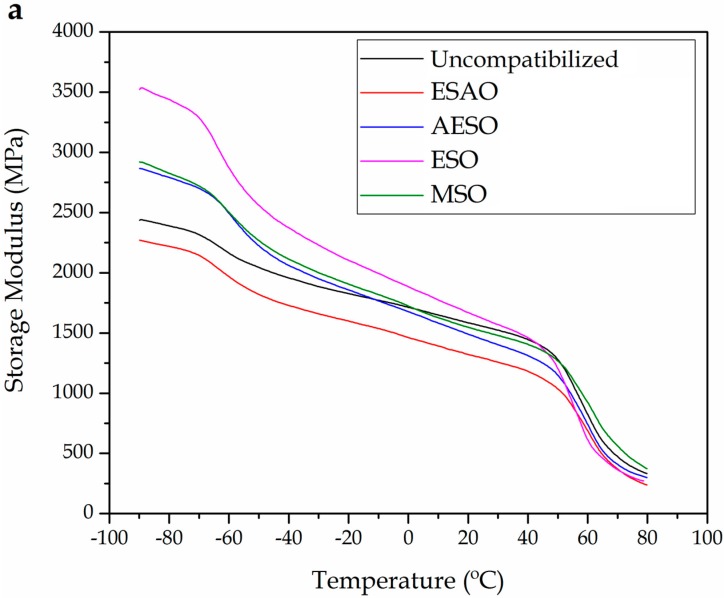

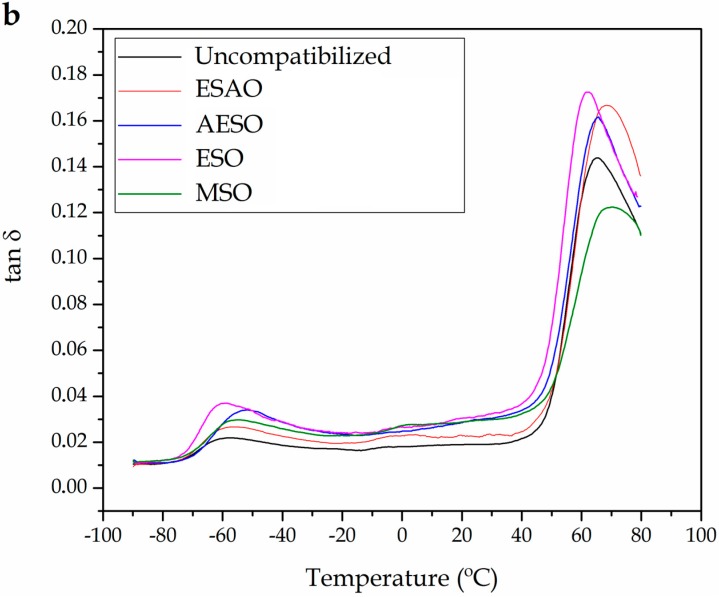
Comparative dynamic-mechanical thermal analysis (DMTA) curves of PLA_60_/PHB_10_/PCL_30_ blend with different compatibilizers, (**a**) storage modulus, E’ and (**b**) damping factor (tan δ).

As has been stated previously, conventional (not modulated) DSC is not sensitive enough to separate some overlapping thermal transitions that occur in the individual components of the blend, and, in consequence, the effects of the different compatibilizers cannot be clearly assessed. For this reason, dynamic-mechanical thermal analysis (DMTA) has been used, as an alternative to measure the characteristic glass transition temperatures of the uncompatibilized and compatibilized blends. The T_g_ for neat PLA is between 55–60 °C while the T_g_ of PHB and PCL is located at −5 and −60 °C, respectively. Figure 5 shows a comparative plot of both the storage modulus (E’) and the damping factor (tan δ) for all uncompatibilized and compatibilized PLA_60_/PHB_10_/PCL_30_ blends. It is possible to observe the T_g_ values of all three PLA, PHB and PCL in the uncompatibilized blend by a step change in the storage modulus curve. Although it is possible to assess the T_g_ value as the onset in the E’ step, it is preferable to define the T_g_ as the peak maximum in the corresponding damping factor curve. Thus, the characteristic T_g_ values are −58, −5 and 65 °C for PCL, PHB and PLA respectively (notice that as PHB represents only a 10 wt% of the total blend, its T_g_ value is remarkably diluted). Addition of 5 phr ESO gives interesting behavior. At low temperatures (from −90 to 20 °C) it gives increased rigidity, but at moderate temperatures (20–80 °C) it seems to be the least stiff material in accordance with previous mechanical characterization. It has a clear effect on the T_g_ values of all three biopolyesters but the main changes are detected for the T_g_ of PCL which changes down to values of −60 °C and the decrease in the T_g_ of PLA down to values of 61.5 °C. This is a clear evidence of the partial plasticization ESO provides to the blend. The other two soybean oil-based compatibilizers, AESO and MSO give similar results in terms of storage modulus and damping factor with temperature. They also show high stiff behavior compared to uncompatibilized blend at low temperatures and more flexible over 0 °C. The changes in T_g_ values that AESO and MSO provide are quite interesting. With regard to AESO, the T_g_ of PCL changes to −50 °C and, similarly, the T_g_ of PLA increases up to 66 °C. This is giving evidences of the branching/crosslinking ability of acrylic compounds in polyester-type polymers as reported by Mauck et al. [37]. With regard to MSO, it gives a T_g_ of PCL at −55 °C and an increased T_g_ of PLA up to 68.5 °C thus indicating its ability to form branch or even crosslinked structures by reaction with the polyester-type polymers in a similar way to acrylic groups. The petroleum-based ESAO compound gives a more flexible behavior compared to uncompatibilized blend for all the temperature range. Due to its crosslinking potential, ESAO gives the highest T_g_ values for PLA-rich phase at about 69.5 °C.

In addition to dynamic mechanical thermal analysis, other thermomechanical properties have been measured to assess the effect of the different compatibilizers. Table 3 summarizes the Vicat softening temperature (VST), heat deflection temperature (HDT) and the coefficient of linear thermal expansion (CLTE) of the blend below the T_g_ of PLA since over this, PCL melts and the obtained results are highly influenced by this process, leading to erroneous values. Regarding VST values, no remarkable changes are detectable, although the blends with AESO and ESAO show slightly higher values than those offered by ESO and MSO, but in any case, the differences are negligible. Regarding HDT values, all compatibilized blends show slightly lower values as flexural conditions are more sensitive to temperature than Vicat test. The highest decrease corresponds to ESO-compatibilized blend. These results are in agreement with previous mechanical results which indicated maximum elongation at break was achieved with 5 phr ESO. Accordingly, the CLTE of the ESO-compatibilized blend reaches the highest values changing from 99.0 µm m^−1^ °C^−1^ (uncompatibilized blend) up to 143.6 µm m^−1^ °C^−1^. The lowest CLTE was obtained for ESAO, accordingly to previous results as this multi-functional oligomer can lead to crosslinking in combination with chain extension and/or polyester compatibilization.

## 3. Materials and Methods

### 3.1. Materials

**Scheme 2:**
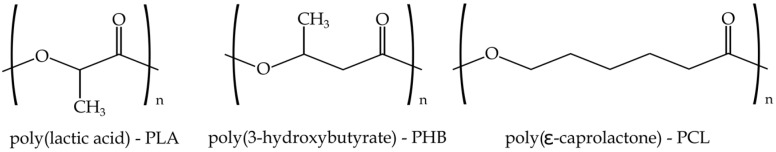
Schematic representation of the chemical structure of the base polyesters for the blend.

The blends were manufactured with PLA, PHB and PCL, whose main properties are summarized in Table 4. Scheme 2 shows their corresponding chemical structures.

**Scheme 3:**
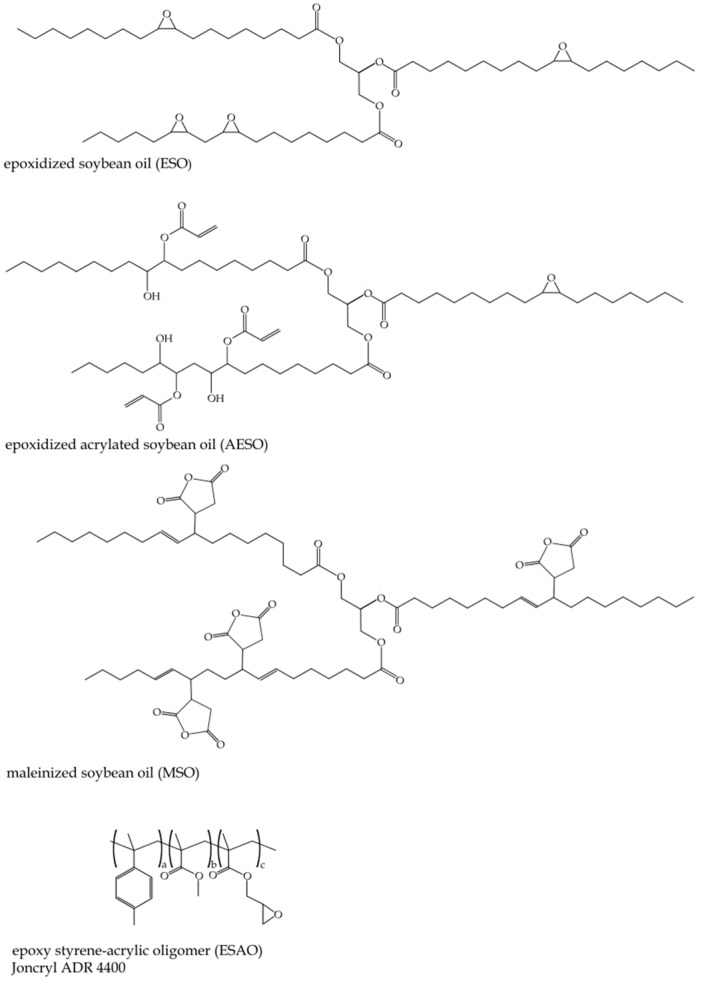
Schematic representation of the chemical structure of compatibilizers used for ternary PLA_60_/PHB_10_/PCL_30_ blends.

Three different soybean oil (SO) derivatives were used as compatibilizers for the blends: epoxidized soybean oil (ESO), maleinized soybean oil (MSO) and acrylated epoxidized soybean oil (AESO). Both ESO and AESO were supplied by Sigma Aldrich S.A. (Madrid, Spain) and were used without any other purification. On the other hand, MSO was synthesized as indicated elsewhere [56], from soybean oil supplied by Gran Velada (Zaragoza, Spain) and maleic anhydride supplied by Sigma Aldrich S.A. (Madrid, Spain). For comparison purposes, a multifunctional epoxy-based styrene-acrylic oligomer (ESAO), Joncryl ADR 4400 from BASF S.A. (Barcelona, Spain), was used. Scheme 3 shows the chemical structure of all four compatibilizers.

### 3.2. Manufacturing of Ternary PLA/PHB/PCL Blends

All three biopolyesters were dehumidified in a MDEO dehumidifier from Industrial Marsé (Barcelona, Spain). PHB and PLA were dried at 60 °C for 24 h while PCL was dried at 45 °C for 24 h. All three vegetable oil derived compatibilizers, ESO, AESO and MSO (in liquid form) were heated to 40 °C to reduce their viscosity and enhance mixing. A constant blend composition was used. Our previous works suggested that a PLA/PHB/PCL blend with a wt% composition of 60/10/30 respectively offers quite balanced properties with improved impact toughness. To evaluate the effects of the three soybean-based compatibilizers, 5 phr (parts of compatibilizer per hundred parts of base blend) of ESO, AESO and MSO were added individually to the base blend. For comparison purposes, an additional formulation containing 1 phr of the petroleum-based compatibilizer, namely ESAO, was used.

The compatibilized blends were manufactured in a twin-screw co-rotating extrusion machine from Construcciones Mecánicas Dupra S.L. (Alicante, Spain). This extruder is characterized by a screw diameter of 25 mm and a length to diameter, L/D ratio of 24. The temperature profile was set to 160, 165, 170 and 175 °C, from the hopper to the extrusion die. The retention time was about 50 s. It is important to remark that biopolyesters, in general, and PHB in particular are highly sensitive to hydrolysis at high temperatures so that, the retention time must not exceed 1 min to avoid further degradation. After cooling, the developed materials were pelletized and further processed by injection moulding in a Sprinter 11 from Erinca S.L. (Barcelona, Spain) equipped with a 18 mm screw diameter. The temperature profile was set to 165 °C (hopper), 170 °C and 175 °C (injection nozzle).

### 3.3. Mechanical Characterization

Tensile properties were obtained in a universal test machine ELIB 50 from S.A.E. Ibertest (Madrid, Spain) as recommended by ISO 527-1:2012. A 5 kN load cell was used and the crosshead speed was set to 5 mm min^−1^. Shore D hardness values were obtained in a 676-D durometer from J. Bot Instruments (Barcelona, Spain) as indicated in ISO 868:2003. The impact strength was obtained using a Charpy’s pendulum (6 J) on notched samples (“V” type notch with a radius of 0.25 mm) as indicated in ISO 179-1:2010. At least, five different measurements were carried out for each formulation and average values of the different properties were calculated. All tests were conducted at room temperature.

### 3.4. Morphology Characterization

The morphology of the fractured samples from impact tests was resolved by field emission scanning electron microscopy (FESEM) in a FESEM microscope ZEISS ULTRA 55 from Oxford Instruments (Abingdon, United Kingdom) working at an accelerating voltage of 2 kV. Samples were subjected to a sputtering process with a gold-palladium alloy in a EMITECH SC7620 sputter-coater from Quorum Technologies, Ltd. (East Sussex, UK). To evaluate the extent of the phase separation, a selective extraction of PCL and PHB was applied with glacial acetic acid supplied by Sigma Aldrich (Madrid, Spain). Fractured samples were immersed in glacial acetic acid solution for 1 h and then, dried and prepared for FESEM observation.

### 3.5. Thermal Characterization

The main thermal properties of the compatibilized blends were obtained by differential scanning calorimetry (DSC) in a Mettler-Toledo 821 (Schwerzenbach, Switzerland) calorimeter. Small pieces with an average weight comprised between 5 and 7 mg were subjected to a temperature program in three steps: initially, a heating stage from −50 up to 200 °C was applied; then, a cooling process down to −50 °C was scheduled and, finally, a second heating ramp from −50 up to 300 °C was programmed. The heating/cooling rate was set to 10 °C min^−1^ for all three steps. Additionally, all stages were run under nitrogen atmosphere (66 mL min^−1^) using standard aluminum crucibles with a capacity of 40 µL.

### 3.6. Thermo-Mechanical Characterization

Dynamic-mechanical thermal analysis (DMTA) was carried out in a DMA1 dynamic analyzer from Mettler-Toledo (Schwerzenbach, Switzerland) working in single cantilever flexural conditions. Samples with dimensions of 10 × 7 × 1 mm^3^, were subjected to a temperature sweep from −90 up to 80 °C at a constant heating rate of 2 °C min^−1^. The selected frequency was 1 Hz and the maximum flexural deformation was set to 10 µm.

Complementary, the Vicat softening temperature (VST) and the heat deflection temperature (HDT) of the uncompatibilized and compatibilized PLA_60_/PHB_10_/PCL_30_ blends were obtained in a Vicat/HDT dual station model VHDT 20 from Metrotec S.A. (San Sebastián, Spain). VST values were obtained using the B50 method with a load of 50 N and a heating rate of 50 °C h^−1^ as recommended in ISO 306. With regard to the HDT measurements, samples with dimensions of 80 × 10 × 4 mm^3^ were placed between supports at a separation of 60 mm. The applied load was calculated as indicated in ISO 75-1 and was 320 g. The heating rate was set to 120 °C h^−1^.

Finally, the dimensional-thermal stability of the developed materials was estimated by thermomechanical analysis (TMA) in a Q400 thermoanalyzer from TA Instruments (Newcastle, DE, USA) with rectangular samples (10 × 10 × 4 mm^3^). A dynamic temperature ramp was programmed from −90 up to 80 °C at a heating rate of 2 °C min^−1^ and a constant load of 0.02 N. All thermal tests were run in triplicate.

## 4. Conclusions

Although the blend from poly(lactic acid) (PLA) (60 wt%), poly(3-hydroxybutyrate (PHB) (10 wt%) and poly(ε-caprolactone) (PCL) (30 wt%) offers improved toughness with regard to neat PLA, the lack (or very poor) miscibility between these three polyesters does not allow optimum results. The use of compatibilizers stands for a technical and cost effective solution to overcome this lack of miscibility. Nevertheless, most of the commercial compatibilizers are petroleum-based additives. This work assesses the potential use of new environmentally friendly additives derived from soybean oil, namely epoxidized soybean oil (ESO), acrylated epoxidized soybean oil (AESO) and maleinized soybean oil (MSO). All three soybean-based compatibilizers have a positive effect on mechanical properties of the blend. In particular, the elongation at break is remarkably improved from 15.3% (uncompatibilized blend) up to values of 130% for the blend with 5 phr ESO. The impact strength is also improved from 5.09 kJ m^−2^ (uncompatibilized blend) up to values of about 11 kJ m^−2^ for blends with MSO and AESO. These soybean oil-based compatibilizers show similar effects to those provided by a commercial multi-functional epoxy styrene-acrylic oligomer, ESAO (Joncryl^®^), widely used as chain extender in polyesters. The use of these vegetable oil-based compatibilizers could represent an environmentally friendly solution to increase compatibility/miscibility in immiscible or partially miscible polyester blends.

## Figures and Tables

**Table 1:** Mechanical properties, tensile modulus (E_t_), tensile strength (σ_t_), elongation at break (ε_b_) hardness and impact strength, of ternary PLA_60_/PHB_10_/PCL_30_ blend with different additives.

Samples	E_t_ (MPa)	σ_t_ (MPa)	ε_b_ (%)	Shore D Hardness	Impact Strength (kJ m^−2^)
Uncompatibilized	2008.8 ± 59.1	48.4 ± 1.32	15.3 ± 1.28	71.8 ± 2.7	5.06 ± 0.94
ESO	1718.3 ± 75.7	34.3 ± 0.89	130.0 ± 3.71	75.2 ± 0.4	9.91± 0.56
AESO	1920.3 ± 63.3	43.2 ± 0.64	45.6 ± 2.35	77.8 ± 0.4	10.87± 0.66
MSO	1521.2 ± 68.3	35.4 ± 1.01	65.8 ± 3.27	74.2 ± 1.7	10.85± 0.61
ESAO	1849.9 ± 78.6	44.5 ± 1.36	44.1 ± 2.15	71.0± 1.8	9.61± 0.48

**Table 2:** Main parameters of the thermal degradation of ternary PLA_60_/PHB_10_/PCL_30_ blend with different compatibilizers, obtained by thermogravimetric analysis.

Sample	T_5_ (°C)	T_max_ (°C)	Residual Weight (%)
Uncompatibilized	308.2 ± 1.13	364.6 ± 1.48	0.48 ± 0.10
ESO	289.1 ± 1.32	345.0 ± 1.62	0.98 ± 0.23
AESO	315.6 ± 1.08	366.5 ± 1.25	0.87 ± 0.12
MSO	309.1 ± 1.41	364.4 ± 1.47	0.83 ± 0.15
Joncryl	311.2 ± 0.96	364.3 ± 1.73	0.54 ± 0.09

T_5_ corresponds to the temperature at which, a weight loss of 5% occurs. T_max_ corresponds to the maximum weight loss rate.

**Table 3:** Thermomechanical properties of ternary PLA_60_/PHB_10_/PCL_30_ blend with different compatibilizers: Vicat softening temperature (VST), heat deflection temperature (HDT) and the coefficient of thermal linear expansion (CLTE) obtained by thermomechanical analysis (TMA).

Sample	VST (°C)	HDT (°C)	CLTE ^1^ (µm m^−1^ °C^−1^)
Uncompatibilized	54.2 ± 0.6	42.3 ± 0.5	99.0 ± 0.42
ESO	53.1 ± 0.3	35.8 ± 0.4	143.6 ± 0.67
AESO	54.2 ± 0.5	37.2 ± 0.6	136.1 ± 0.89
MSO	53.7 ± 0.5	40.6 ± 0.4	127.9 ± 0.62
ESAO	54.3 ± 0.4	36.2 ± 0.3	102.3 ± 0.85

^1^ The CLTE was measured in the temperature range comprised between 10 °C and 50 °C.

**Table 4:** Summary of the commercial grade polymers used for blends manufacturing and some of their relevant properties.

Polymer	Grade	Supplier	Density (g cm^−3^)	T_g_ (°C)	T_m_ (°C)	MFI (g/10 min)
Poly(lactic acid)–PLA	Ingeo™ Biopolymer 6201D	NatureWorks	1.240	55–60	155–170	15–30 @ 210 °C
Poly(3-hydroxybutyrate)–PHB	P226	Biomer	1.250	−5	170	10 @ 180 °C
Poly(ε-caprolactone)–PCL	Capa TM6800	Perstorp UK Ltd	1.146	−50/−60	58-60	2.01–4.03 @ 160 °C
